# Impact of Tricuspid Regurgitation on the Clinical Outcomes of Patients with Heart Failure

**DOI:** 10.1002/puh2.70092

**Published:** 2025-09-17

**Authors:** Muhammad Usman Almani, Rasha Khan, Noor Fatima, Muhammad Yousuf, Aman Amanullah

**Affiliations:** ^1^ Division of Cardiology Jefferson Einstein Hospital Thomas Jefferson University Philadelphia Pennsylvania USA; ^2^ Division of Internal Medicine Jefferson Einstein Hospital Thomas Jefferson University Philadelphia Pennsylvania USA; ^3^ Division of Medicine Nishtar Medical University Multan Punjab Pakistan; ^4^ Division of Medicine Mayo Clinic Rochester Minnesota USA

**Keywords:** gender differences, heart failure, socioeconomic disparities, tricuspid regurgitation

## Abstract

**Background:**

Tricuspid regurgitation (TR) is a common occurrence in patients with heart failure (HF), and its role in disease progression has gained attention in recent years. Although TR can worsen clinical outcomes in HF patients, the impact of gender, racial, and socioeconomic factors remains largely unexplored. With growing evidence supporting the role of percutaneous interventions for the treatment of significant TR, understanding these disparities is more crucial than ever.

**Methods:**

Data were extracted from the National Inpatient and National Readmission 2016–2020 Databases. We used ICD‐10 code I50 to identify the patients primarily admitted for HF and subdivided the cohort into two groups based on the presence or absence of TR. We performed multivariable logistic regression analysis to determine odds of the in‐hospital mortality and multivariable Cox regression analysis to assess the 30‐ and 90‐day hospital readmission in HF patients with and without TR. All the analyses were adjusted for age, gender, insurance status, Charlson comorbidity index, and hospital characteristics. STATA 16 software was used for analysis.

**Results:**

There was no difference in the in‐hospital mortality among HF patients with and without TR (OR: 1.04, 95% CI 0.94–1.16, *p* = 0.442) except in certain subgroups of HF patients. HF patients with TR were 6% more likely to have HF‐specific readmission in 30 days (HR: 1.06, 95% CI 1.00–1.13, *p* = 0.044) and 9% more likely to have HF‐specific readmission in 90 days (HR: 1.09, 95% CI 1.03–1.15, *p* = 0.002). Subgroup analysis revealed significant gender, racial, and socioeconomic disparities in the in‐hospital mortality and the readmission outcomes of HF patients with TR compared to those without TR.

**Conclusion:**

In our population‐based survey analysis, we observed significant gender, racial, and socioeconomic disparities in the clinical outcomes of HF patients with TR compared to those without TR.

## Graphical abstract

There are significant gender, racial, and socioeconomic disparities in the clinical outcomes of heart failure (HF) patients with tricuspid regurgitation (TR) when compared to those without TR.

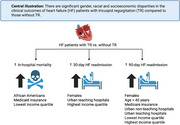

## 1 Introduction

Heart failure (HF) remains a significant global health burden, affecting millions of individuals worldwide and contributing substantially to healthcare costs and mortality rates. The lifetime risk of HF in the United States is approximately 24%; approximately 6.7 million Americans over 20 years of age have HF, and the prevalence is expected to rise to 8.5 million Americans by 2030 [1]. Within this context, tricuspid regurgitation (TR) has emerged as a comorbidity that can significantly impact the prognosis and management of HF patients. TR is more common in patients with HF as compared to general population, and the TR is being increasingly recognized as more than just a benign consequence of left‐sided heart disease [2]. Moderate or severe TR was observed in about 20% of patients with chronic HF in the European Society of Cardiology Heart Failure Long‐Term Registry regardless of left ventricular ejection fraction [3].

Recent studies have highlighted the independent prognostic significance of TR in patients with HF. Moderate‐to‐severe TR has been associated with increased mortality and hospitalization rates, even after adjusting for other cardiovascular risk factors [4]. Despite these findings, the specific impact of TR on readmission rates and the potential gender and socioeconomic disparities in outcomes remain areas of active investigation.

The relationship between TR and HF is complex and multifaceted. TR can occur as a primary valvular disorder or, more commonly, as a secondary consequence of right ventricular dilation and dysfunction in the setting of left‐sided heart disease or pulmonary hypertension. The presence of TR in HF patients can lead to a vicious cycle of worsening right ventricular function, increased right atrial pressure, and further TR progression, potentially contributing to more frequent hospitalizations and poor outcomes [5].

Gender differences in cardiovascular disease presentation, progression, and outcomes have been increasingly recognized in recent years. However, the specific impact of gender on the interplay between TR and HF outcomes, particularly regarding in‐hospital mortality and readmission rates, has not been thoroughly explored. Understanding these potential disparities is key for developing targeted interventions and providing patient‐centered care. Socioeconomic factors also influence HF outcomes, with studies indicating a correlation between lower income and higher readmission rates [6]. Nevertheless, the relationship between socioeconomic status and TR in HF outcomes warrants further examination.

Our findings support the development of targeted management strategies for HF patients with TR and highlight the importance of considering gender and socioeconomic factors in cardiovascular care. With growing evidence supporting the role of percutaneous interventions for the treatment of significant TR, understanding these disparities is more crucial than ever in order to provide equitable and patient‐centered care. The aim of this study is to evaluate the impact of TR on in‐hospital mortality and readmission outcomes in the HF patients, with a specific focus on gender and socioeconomic disparities in these clinical outcomes.

## 2 Methods

### 2.1 Design and Data Source

This was a retrospective analysis involving adult hospitalizations for HF in the United States from 2016 to 2020. We extracted the data from the National Inpatient Sample (NIS) and National Readmission Database (NRD). We used the NIS for patients’ characteristics analysis and in‐hospital outcomes and the NRD for readmission analysis. The NIS and NRD are part of the Healthcare Cost and Utilization Project (HCUP) that is sponsored by the Agency for Healthcare Research and Quality (AHRQ). The NIS is the largest all‐payer database of hospital inpatient stays in the United States. However, the NRD is drawn from HCUP State Inpatient Databases (SID) that contain reliable, verified patient linkage numbers that can be used to track a person across hospitals within a state, while adhering to strict privacy guidelines. Each discharge information includes de‐identified elements, such as patient demographics, payment source, hospital characteristics, principal diagnosis, secondary diagnoses, and procedural diagnoses, among others. The NIS and NRD contain a weighted sample of hospitalizations, and this can be used to derive national estimates. The study was exempt from institutional board review approval, as the NIS and NRD databases contain de‐identified patient information.

### 2.2 Study Population

Eligible patients for this study included US adults aged ≥18 years who were admitted with a principal diagnosis of HF between 2016 and 2020. We used International Classification of Diseases, 10th Revision, Clinical Modification (ICD‐10‐CM) code I50 to identify patients primarily admitted for HF. The ICD‐10‐CM code I50 encompasses diastolic HF, systolic HF, combined diastolic and systolic HF, and biventricular HF. No additional patients were excluded for in‐hospital outcome analysis using NIS database. For readmission analysis using NRD database, admissions were excluded as an index admission if the hospitalization was elective, had missing data for age, sex, or in‐hospital mortality, or if the patient died during the hospital stay or was transferred to another acute care hospital. In the NRD, patient identifiers cannot be linked across the years; hence, patients who had an index hospitalization on December 1 or later in any given year were excluded for 30‐day readmission analysis, and patients who had an index hospitalization on October 1 or later in any given year were excluded for 90‐day readmission analysis. Time to readmission was calculated by subtracting the length of stay of index admission from time between index admission and the readmission. Planned/Elective readmissions were excluded. Readmissions for nonspecific traumatic diagnoses were excluded using the NECODE. The NECODE provides a method of classifying injuries. The NECODE used for nonspecific traumatic readmission exclusion was ICD‐10 codes, which are “S, T, V, and Y.”

### 2.3 Variables

Patient demographics, including age, sex, primary insurance, race, and median neighborhood household income (income quartiles were identified and referred to patients as 1—low income, 2—middle income, 3—upper middle income, and 4—high income), were obtained using the NIS and NRD variables. In 2020, Quartile 1 reflected neighborhood household income: ≤$49,999; Quartile 2: $50,000–$64,999; Quartile 3: $65,000–$85,999; and Quartile 4: ≥$86,000. The expected primary payer was identified as one of the following: Medicaid, Medicare, or private insurance. In the United States, Medicaid is a joint federal and state program that helps cover medical costs for some people with limited income and resources, whereas Medicare is a federal health insurance program for people aged 65 or older. We included hospital‐specific variables, including hospital bed size (small, medium, or large), hospital teaching status (teaching hospital or non‐teaching hospital), hospital location (rural or urban), and hospital region (Northeast, Midwest, South, or West). Comorbidities were identified using diagnoses codes from the ICD‐10‐CM respective to years (Table S1). The co‐morbid conditions were present prior to the hospitalization and were not the primary cause of admission. We used the Charlson comorbidity index (CCI) to assess the severity of co‐morbid conditions.

### 2.4 Outcome Measures

In the primary analysis, in‐hospital mortality for patients primarily admitted for HF was assessed. A secondary analysis assessed unplanned (i.e., non‐elective) readmission occurring within the first 30 and 90 days of discharge from the index hospitalization. If an index hospitalization had more than one readmission within 30 and 90 days, we only included the first readmission for the 30‐ and 90‐day analysis, respectively.

### 2.5 Statistical Analysis

As per specific HCUP recommendations, we performed all our analysis utilizing the HCUP STATA survey data analysis packages. The Student's *t*‐test and the chi‐squared test were used to compare continuous and categorical variables, respectively.

We performed multivariable logistic regression analysis to determine odds ratio of in‐hospital mortality in HF patients with and without TR. Subgroup analyses were performed on the basis of (1) gender, (2) age, (3) race, (4) insurance status, (5) location and teaching status of admitting hospital, and (6) patients’ neighborhood household income quartile. The basic assumptions for logistic regression model include independence of errors, absence of multicollinearity, and lack of strongly influential outliers.

In the secondary analysis we performed multivariable Cox regression analysis with HF‐specific readmission within 30 and 90 days as the “failure event” and time from index hospitalization discharge to failure event as the “time to failure event”. HF‐specific readmission was defined as the HF being the primary diagnosis for readmission. Time to failure event was calculated by subtracting the length of stay of index admission from time between index admission and the failure event. Subgroup analyses were performed on the basis of (1) gender, (2) age, (3) insurance status, (4) location and teaching status of admitting hospital, and (5) patients’ neighborhood household income quartile. The basic assumptions for the Cox regression model include that observations should be independent and the hazard ratios should be constant across time.

All the analyses were adjusted for age, sex, insurance status, CCI, and hospital characteristics (bed size, location). A 2‐sided *p* < 0.05 was considered to represent statistical significance. All analyses were performed using STATA version 16.

## 3 Results

### 3.1 Prevalence of TR Among HF Hospitalizations

Among 1,365,529 hospitalizations for a primary diagnosis of HF, 58,308 (4.27%) had a secondary diagnosis of TR.

### 3.2 Baseline Characteristics

Baseline patient and hospital characteristics of HF hospitalizations with and without TR are presented in Table 1. The mean age of HF patients with TR was 71.3 years, mean CCI was 3.4, and 55.8% of the patients were female. In contrast, mean age of HF patients without TR was 70.7 years, mean CCI was 3.5, and 47.5% of the patients were female. HF patients with TR had longer mean lengths of stay, and they were more likely to have pulmonary hypertension, the presence of a defibrillator, a pacemaker, or a prosthetic heart valve; however, they were less likely to have hypertension, diabetes mellitus, or obesity.

**TABLE 1:** Baseline characteristics of heart failure patients with and without tricuspid regurgitation.

Baseline characteristics	Without TR (*n* = 1,307,221)	With TR (*n* = 58,308)	*p* value
Age (years), mean	70.7	71.3	<0.001
Charlson comorbidity index, mean	3.5	3.4	0.006
LOS (days), mean	5.2	6.4	<0.001
Weekend admission, *n* (%)	303,275 (23.2)	12,565 (21.6)	<0.001
Female, *n* (%)	620,276 (47.5)	32,548 (55.8)	<0.001
Race, *n* (%)			<0.001
Caucasians	922,506 (70.6)	39,539 (67.8)	
African Americans	224,058 (17.1)	11,283 (19.4)	
Hispanics	97,257 (7.4)	4297 (7.4)	
Median household income, *n* (%)			<0.001
Low‐income quartile	436,873 (33.4)	18,437 (31.6)	
Middle‐income quartile	357,002 (27.3)	14,863 (25.5)	
Upper‐middle‐income quartile	294,648 (22.5)	13,790 (23.7)	
High‐income quartile	218,567 (16.7)	11,213 (19.2)	
Insurance, *n* (%)			<0.001
Medicare	920,675 (70.4)	41,539 (71.2)	
Medicaid	151,899 (11.6)	7137 (12.2)	
Private insurance	166,017 (12.7)	7073 (12.1)	
Hospital location and teaching status, *n* (%)			<0.001
Rural	198,567 (15.2)	6076 (10.4)	
Urban non‐teaching	332,034 (25.4)	12,781 (21.9)	
Urban teaching	776,489 (59.4)	39,451 (67.7)	
Hospital region, *n* (%)			<0.001
Northeast	242,882 (18.6)	10,775 (18.5)	
Midwest	305,759 (23.4)	14,868 (25.5)	
South	506,025 (38.7)	21,131 (36.2)	
West	252,555 (19.3)	11,533 (19.8)	
Hospital bed size, *n* (%)			<0.001
Small	304,975 (23.3)	11,907 (20.4)	
Medium	370,989 (28.4)	15,341 (26.3)	
Large	631,126 (48.3)	31,055 (53.3)	
Hypertension, *n* (%)	823,419 (63.0)	35,755 (61.3)	0.002
Dyslipidemia, *n* (%)	569,033 (43.5)	24,839 (42.6)	0.058
Diabetes mellitus, *n* (%)	538,575 (41.2)	19,632 (33.7)	<0.001
Obesity, *n* (%)	282,883 (21.6)	10,840 (18.6)	<0.001
Peripheral arterial disease, *n* (%)	88,760 (6.8)	3743 (6.4)	0.132
History of ischemic stroke, *n* (%)	5621 (0.4)	222 (0.4)	0.368
Smoking, *n* (%)	186,279 (14.3)	7341 (12.6)	<0.001
COPD, *n* (%)	453,083 (34.7)	17,976 (30.8)	<0.001
Major depressive disorder, *n* (%)	144,709 (11.1)	6373 (10.9)	0.642
Alcoholism, *n* (%)	52,158 (4.0)	2531 (4.3)	0.070
Protein energy malnutrition, *n* (%)	51,505 (3.9)	3020 (5.2)	<0.001
Anemia, *n* (%)	406,677 (31.1)	21,347 (36.6)	<0.001
Pulmonary hypertension, *n* (%)	257,915 (19.7)	30,723 (52.7)	<0.001
Presence of pacemaker, *n* (%)	120,264 (9.2)	7353 (12.6)	<0.001
Presence of ICD, *n* (%)	136,997 (10.5)	6501 (11.2)	0.027
Prior MI, *n* (%)	178,044 (13.6)	6945 (11.9)	<0.001
Prior percutaneous coronary intervention, *n* (%)	136,997 (10.5)	4974 (8.5)	<0.001
Prior coronary artery bypass grafting, *n* (%)	175,168 (13.4)	7254 (12.4)	0.003
Presence of prosthetic valve, *n* (%)	66,538 (5.1)	4752 (8.2)	<0.001

Abbreviations: ICD, implantable cardioverter defibrillator; LOS, length of stay; COPD, chronic obstructive pulmonary disease; MI, myocardial infarction; TR, tricuspid regurgitation.

### 3.3 In‐Hospital Mortality

**FIGURE 1:**
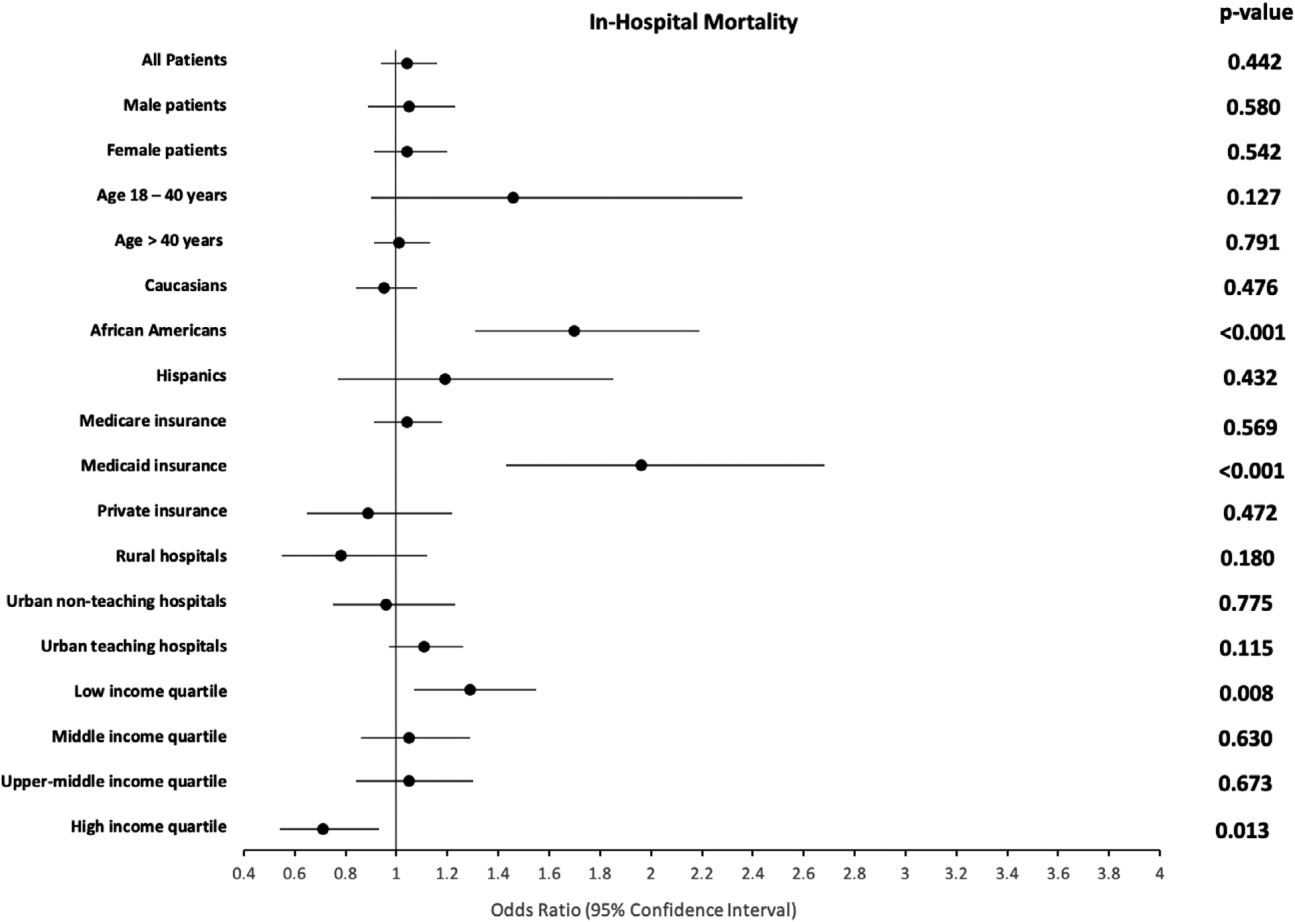
Impact of TR on in‐hospital mortality among HF patients (reference group: patients without TR). In 2020, low‐income quartile reflected household income: ≤$49,999; middle‐income quartile: $50,000–$64,999; upper‐middle‐income quartile: $65,000–$85,999; high‐income quartile: ≥$86,000. HF, heart failure; TR, tricuspid regurgitation.

Among 1,365,529 HF patients, 41,177 (3.15%) patients without TR and 1965 (3.37%) patients with TR died during the hospitalization. In the adjusted analysis, there was no difference overall in in‐hospital mortality of HF patients with and without TR (OR: 1.04, 95% CI 0.94–1.16, *p* = 0.442). However, in subgroup analysis, African Americans had a 70% higher risk of in‐hospital mortality (OR: 1.70, 95% CI 1.31–2.19, *p* < 0.001), patients with Medicaid insurance had a 96% higher risk of in‐hospital mortality (OR: 1.96, 95% CI 1.43–2.68, *p* < 0.001), and patients from the lowest neighborhood household income quartile had 29% higher risk of in‐hospital mortality (OR: 1.29, 95% CI 1.07–1.55, *p* = 0.008) when compared to their counterparts without TR, as shown in Figure 1.

### 3.4 Thirty‐Day HF‐Specific Readmission

**FIGURE 2:**
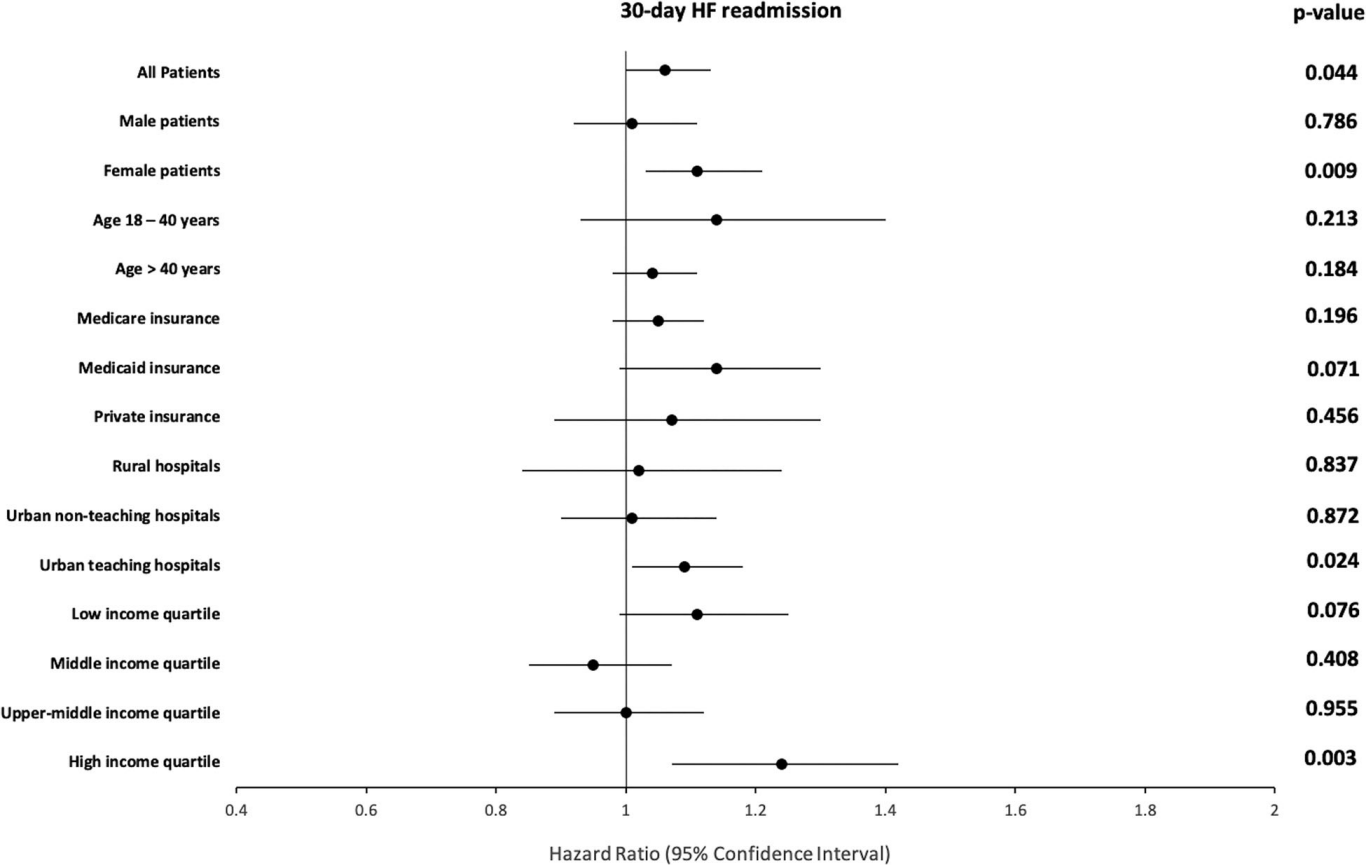
Impact of TR on 30‐day HF‐specific readmission among HF patients (reference group: patients without TR). In 2020, low‐income quartile reflected household income: ≤$49,999; middle‐income quartile: $50,000–$64,999; upper‐middle‐income quartile: $65,000–$85,999; high‐income quartile: ≥$86,000. HF, heart failure; TR, tricuspid regurgitation.

Among 1,162,314 HF patients assessed for 30‐day readmission analysis, 59,764 (5.37%) patients without TR and 2771 (5.61%) patients with TR had a 30‐day HF‐specific readmission. In the adjusted analysis, HF patients with TR were 6% more likely to have HF‐specific readmission in 30 days (HR: 1.06, 95% CI 1.00–1.13, *p* = 0.044). In the subgroup analysis, female patients had an 11% higher risk of 30‐day HF‐specific readmission (HR: 1.11, 95% CI 1.03–1.21, *p* = 0.009), patients admitted to the urban teaching hospitals had a 9% higher risk of 30‐day HF‐specific readmission (HR: 1.09, 95% CI 1.01–1.18, *p* = 0.024), and patients from the highest neighborhood household income quartile had 24% higher risk of 30‐day HF‐specific readmission (HR: 1.24, 95% CI 1.07–1.42, *p* = 0.003) when compared to their counterparts without TR, as shown in Figure 2.

### 3.5 Ninety‐Day HF‐Specific Readmission

**FIGURE 3:**
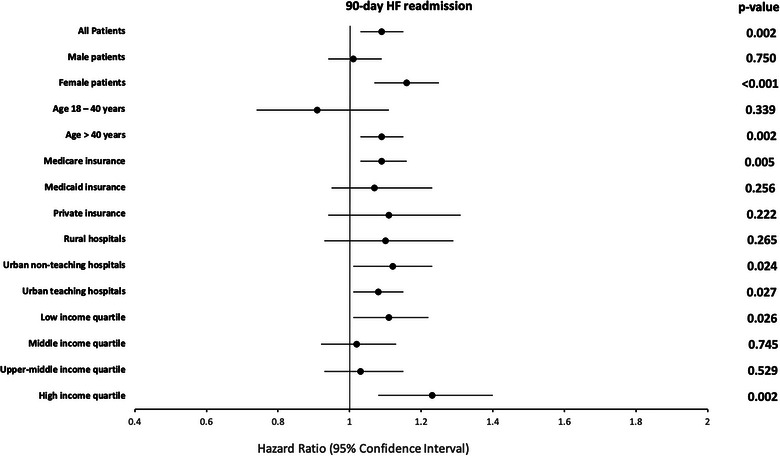
Impact of TR on 90‐day HF‐specific readmission among HF patients (reference group: patients without TR). In 2020, low‐income quartile reflected household income: ≤$49,999; middle‐income quartile: $50,000–$64,999; upper‐middle‐income quartile: $65,000–$85,999; high‐income quartile: ≥$86,000. HF, heart failure; TR, tricuspid regurgitation.

Among 971,368 HF patients assessed for 90‐day readmission analysis, 83,987 (9.03%) patients without TR and 3988 (9.66%) patients with TR had a 90‐day HF‐specific readmission. In the adjusted analysis, HF patients with TR were 9% more likely to have HF‐specific readmission in 90 days (HR: 1.09, 95% CI 1.03–1.15, *p* = 0.002). In the subgroup analysis, female patients had 16% higher risk of 90‐day HF‐specific readmission (HR: 1.16, 95% CI 1.07–1.25, *p* < 0.001), patients with age >40 years had 9% higher risk of 90‐day HF‐specific readmission (HR: 1.09, 95% CI 1.03–1.15, *p* = 0.002), patients with Medicare insurance had 9% higher risk of 90‐day HF‐specific readmission (HR: 1.09, 95% CI 1.03–1.16, *p* = 0.005), patients admitted to urban non‐teaching hospitals had 12% higher risk of 90‐day HF‐specific readmission (HR: 1.12, 95% CI 1.01–1.23, *p* = 0.024), patients admitted to urban teaching hospitals had 8% higher risk of 90‐day HF‐specific readmission (HR: 1.08, 95% CI 1.01–1.15, *p* = 0.027), patients from the lowest neighborhood household income quartile had 11% higher risk of 90‐day HF‐specific readmission (HR: 1.11, 95% CI 1.01–1.22, *p* = 0.026), and patients from the highest neighborhood household income quartile had 23% higher risk of 90‐day HF‐specific readmission (HR: 1.23, 95% CI 1.08–1.40, *p* = 0.002) when compared to their counterparts without TR, as shown in Figure 3.

## 4 Discussion

The greatest strength of our study is the breadth and longevity of the data captured in the NIS and the NRD databases. These databases are derived from a large and diverse population that is representative of the entire United States. The large sample size allowed us to achieve greater precision in the estimation, thereby providing more reliable results.

Studies regarding the influence of gender and socioeconomic status on TR outcomes in HF patients are lacking. Our study provides important insights into the relationship between TR and HF patients regarding inpatient mortality and short‐term readmission outcomes, with a particular focus on gender differences and socioeconomic factors. The key findings of our research highlight several important patterns that align with and extend the current understanding in the field. We found that certain subgroups of HF patients with TR (African Americans, patients with Medicaid insurance, and patients from the low neighborhood household income quartile) had a higher risk of in‐hospital mortality. These findings align with a large electronic health record analysis, which demonstrated that both prevalent and incident TR were independently associated with a higher risk of death in certain subgroups of HF patients [7]. Additionally, our results indicate that patients with HF and concomitant TR were 6% more likely to have HF‐specific readmission within 30 days and 9% more likely to have HF‐specific readmission within 90 days compared to HF patients without TR. This finding is consistent with previous studies that have demonstrated TR as an independent risk factor for poor outcomes in HF patients. Studies have shown that severe TR independently increases the risk of recurrent HF hospitalizations and that significant TR is linked to higher rates of HF readmissions [7, 8, 9].

A notable finding of our study is the higher 30‐ and 90‐day readmission rates observed in female patients with HF and TR. This gender disparity in outcomes is a critical area that requires further investigation. Our findings contribute to a growing body of evidence highlighting gender‐based differences in TR prevalence and outcomes. The increased readmission rates observed can be attributed to a complex interplay of biological, anatomical, and clinical factors. Epidemiological studies consistently report a higher prevalence of TR in women, with over 60% of patients with more than moderate TV regurgitation being female [10, 11]. This gender disparity may be partly explained by anatomical differences, as El‐Busaid et al. demonstrated in 2012 that female atrioventricular valve annuli are less elastic and have a more scattered cellular distribution within the collagen matrix compared to males, potentially predisposing women to valvular incompetence [12]. Hormonal factors also play a crucial role, with estrogen influencing cardiovascular remodeling and electrophysiological properties. This hormonal influence may contribute to the higher rates of atrial fibrillation and HF with preserved ejection fraction (HFpEF) observed in women with TR, conditions that increase right‐sided pressures and lead to right atrial and ventricular dilation [13, 14]. Furthermore, the clinical management of TR in women is complicated by delayed diagnosis and intervention. Women often present with more advanced disease due to undiagnosed and insidious progression of atrial secondary TR, leading to late right ventricular dysfunction [14]. This delay is compounded by current valvular guidelines, which do not account for the typically smaller cardiac dimensions in women, potentially resulting in inadequate diagnosis and delayed referral for intervention. Collectively, these factors create a confluence of events that may explain the higher readmission rates observed in female patients with TR. Our results highlight the need for enhanced post‐discharge monitoring, particularly for women with HF and TR, to prevent early readmissions. Close monitoring may help detect and manage complications early, potentially averting the need for rehospitalization. The gender differences in readmission rates suggest a need to consider gender‐specific approaches in patient care. Potential strategies may include adjusting screening timelines for TR in women and developing treatment plans that consider gender‐specific risk factors. These approaches could contribute to improved outcomes for female patients with HF and TR.

Additionally, our findings emphasize the importance of addressing TR in the overall management of HF patients. This is supported by recent research from Zancanaro et al., which demonstrated that treatment of TR in patients with right‐sided HF significantly reduced cardiac death and HF hospitalization rates at 1 year [15]. These results suggest that a more aggressive approach to TR management, possibly including tricuspid valve interventions, could effectively reduce readmission rates in HF patients. Importantly, our study revealed a higher risk of inpatient mortality among HF and TR patients with specific socioeconomic characteristics, particularly for African Americans, patients with Medicaid insurance, and patients from the lower neighborhood household income quartiles. These disparities appear to stem from multiple factors. Within the African American demographic, epidemiologic studies have established associations with poorer clinical outcomes, likely due to increased risk factors, comorbidities, and a component of genetic predisposition to cardiac disease burden. This may be further implicated by limitations in healthcare accessibility and household income, as suggested by our findings and additionally supported by previous research on socioeconomic disparities in HF outcomes [6]. For instance, Medicaid's low reimbursement rates, limited provider networks, and restrictive coverage often delay care and reduce access to specialists, whereas socioeconomic barriers such as housing instability, food insecurity, and lack of transportation further contribute to worse clinical outcomes in these populations. Interestingly, our analysis revealed a contrasting pattern of increased 30‐ and 90‐day hospital readmissions observed among patients from the higher neighborhood household income quartiles. This paradox may be explained by greater healthcare utilization and access in this population, driven by heightened health literacy and recognition of concerning symptoms. Additionally, comprehensive insurance coverage may influence providers’ decisions to readmit these patients more readily.

Our study results collectively emphasize the need for equitable and patient‐centered interventions for HF patients with TR that address both clinical and socioeconomic aspects of patient care. Integrating enhanced monitoring, gender‐specific interventions, effective TR management, and support for disadvantaged populations can lead to more personalized care for HF patients with TR. Policies addressing the gender and socioeconomic disparities in this patient population are needed to reduce readmissions, lower mortality rates, and improve overall clinical outcomes.

## 5 Limitations

This is a retrospective evaluation of a large administrative database, so causal inference cannot be established, and unmeasured confounders may exist. The database did not accurately characterize the severity of TR (mild, moderate, or severe), which could have provided further insights into the relationship of TR on the clinical outcomes of patients with HF. We excluded December admissions for the 30‐day readmission analysis and October‐to‐December admissions for the 90‐day readmission analysis; hence, our analysis does not account for readmission outcomes of patients during these months. The data regarding guideline‐directed medical therapy for HF and New York Heart Association classification are not available in the NRD; hence, the impact of these factors on readmission outcomes could not be assessed. NRD does not contain data regarding the race of the patients; hence, the interplay among race, TR, and HF readmissions could not be determined.

## 6 Conclusions

Our study demonstrates the significant impact of TR on in‐hospital mortality and readmission rates in HF patients, revealing important gender disparities and socioeconomic influences. The findings highlight the need for targeted management strategies, particularly for female patients and those from lower socioeconomic backgrounds. This warrants further investigation to clarify the underlying mechanisms of these disparities and to develop and evaluate tailored interventions, including more aggressive TR management strategies that address disparities among HF patients with co‐morbid TR.

## Author Contributions

Muhammad Usman Almani and Rasha Khan contributed to the analysis of data and drafting the manuscript. Noor Fatima and Muhammad Yousuf contributed to conception of the study and interpretation of data. Aman Amanullah contributed to validation, supervision and making substantial revisions to the study manuscript.

## Ethics Statement

The authors have nothing to report.

## Consent

The authors have nothing to report.

## Conflicts of Interest

The authors declare no conflicts of interest.

## Guarantor Statement

All the authors take responsibility for the content of the manuscript, including the data and analysis.

## Supporting information




**Supporting Table 1:** Diagnosis Codes Utilized in Study. COPD, chronic obstructive pulmonary disease; ICD, implantable cardioverter‐defibrillator.

## Data Availability

The National Inpatient Sample (NIS) and National Readmission Database (NRD) are readily and publicly available. The patient information is de‐identified, thus exempting the studies from the IRB process. URL: https://hcup‐us.ahrq.gov/databases.jsp.
