# Supplementary material for: Impact of Tricuspid Regurgitation on the Clinical Outcomes of Patients with Heart Failure
Source: Public Health Chall. 2025 Sep 17;4(3):e70092. doi: 10.1002/puh2.70092 (PMC12442787; doi:10.1002/puh2.70092)
Supplement: Supplementary file 1 — Supporting Table 1: Diagnosis Codes Utilized in Study. COPD, chronic obstructive pulmonary disease; ICD, implantable cardioverter‐defibrillator. [file PUH2-4-e70092-s001.docx]

|  | |  |
| --- | --- | --- |
| Variables | ICD-10 CM/PCS codes | |
| Tricuspid regurgitation | I361, I071 | |
| Heart Failure | I501, 15020, 15021, 15022, 15023, 15030, 15031, 15032, 15033, 15040, 15041, 15042, 15043, 150810, 150811, 150812, 150813, 150814, 15082, 15083, 15084, 15089, 1509 | |
| Hypertension | I10, I110, I119, I120, I129, I130, I1310, I1311, I132, I150, I151, I152, I158, I159 | |
| Diabetes mellitus | E08 - E13 | |
| Obesity | E66x | |
| Dyslipidemia | E78x | |
| Peripheral arterial disease | I739 | |
| Smoking | F17x | |
| COPD | J41x, J42x, J43x, J44x | |
| Pulmonary hypertension | I270, I272 | |
| Ischemic stroke | I63 | |
| Major depressive disorder | F32x, F33x | |
| Alcoholism | F10x | |
| Protein energy malnutrition | E43, E440, E441 | |
| Anemia | D50x, D51x, D52x, D53x, D54x, D55x, D56x, D57x, D58x, D59x, D60x, D61x, D62x, D63x, D64x | |
| Presence of pacemaker | Z950 | |
| Presence of ICD | Z95810 | |
| Prior coronary artery bypass grafting | Z951 | |
| Prior percutaneous coronary intervention | Z955 | |
| Prior myocardial infarction | I252 | |
| Presence of prosthetic valve | Z952, Z953 | |

**Supplemental Table 1. Diagnosis Codes Utilized in Study.** COPD: Chronic obstructive pulmonary disease, ICD: Implantable cardioverter defibrillator.
